# Evaluating the “wrong-way-round” electrospray ionization of antiretroviral drugs for improved detection sensitivity

**DOI:** 10.1007/s00216-022-04499-1

**Published:** 2023-01-13

**Authors:** Pieter Venter, Rianita van Onselen

**Affiliations:** Biomedical Research and Innovation Platform, South African Medical Research Council, P.O. Box 19070, Tygerberg, 7505 South Africa

**Keywords:** Antiretroviral drugs, Mass spectrometry, “Wrong-way-round” electrospray ionization, Mixed-mode strong cation exchange sorbent, Mixed-mode strong anion exchange sorbent

## Abstract

The presence of antiretroviral drugs (ARVDs) in the aquatic environment poses a significant health risk to the ecosystem. The dilution of these compounds during wastewater treatment processes, followed by discharge into the environment, results in extremely low concentrations in the range of ng/L. Therefore, to enable detection of these low concentrations, it is important to determine the most efficient electrospray ionization (ESI) mode using the right mobile phase modifier and to establish a selective extraction procedure. In this study, we compared the ESI intensity in the positive and negative mode using both formic acid (FA) and ammonium hydroxide (NH_4_OH) as mobile phase modifiers. The results revealed a phenomenon known as the “wrong-way-round” (WWR) ESI in which high intensity [M + H]^+^ ions were detected under basic conditions using NH_4_OH as modifier and, similarly, high intensity [M-H]^−^ ions were detected under acidic conditions using FA as modifier. Furthermore, mixed-mode strong cation (MCX) and mixed-mode strong anion (MAX) exchange sorbents were evaluated for extraction recoveries, which yielded extraction recoveries between 60 and 100%. Finally, the recoveries obtained using mixed-mode ion exchange sorbents compared to ion production during the ESI process provide evidence that ions produced in solution do not necessarily reflect the ions that are produced during the ESI process. Based on the results of this study, it is recommended to evaluate the optimal ionization mode under basic and acidic conditions, instead of defaulting to the use of acidic modifiers with positive ion detection.

**Supplementary Information:**

The online version contains supplementary material available at 10.1007/s00216-022-04499-1.

## Introduction

The accurate quantification of ARVDs present in wastewater effluent and environmental surface water has become a necessity because of the potential risks that continuous and inadvertent low-level exposure poses to non-targeted organisms [1, 2] and the potential development of ARVD-resistant HIV-1 strains through unknown, low-level exposure to ARVDs in drinking water and food sources [3]. Liquid chromatography (LC) coupled with tandem mass spectrometry (MS/MS) equipped with an ESI source is the preferred method for quantifying ARVDs in aqueous samples [4]. Typically, detection of ARVDs is predominantly in the positive ion [M + H]^+^ mode using ammonium formate, acetate, or formic acid as mobile phase modifiers [4]. The reason for predominantly using the positive ion mode is based on the premise that ARVDs contain weakly basic functional groups, i.e., amides with favorable protonation on the oxygen [5–7] and primary and secondary amines with preferred protonation on the nitrogen [8] in an acidic solution. In addition, it is generally assumed that the charge of the ion produced during the electrospray process depends on the mobile phase pH [9]. However, it has been shown that high intensity [M + H]^+^ ions can be produced under strongly basic LC conditions and, similarly, that high intensity [M-H]^−^ ions can be produced under strongly acidic LC conditions. This interesting ionization behavior was first observed by Kelly et al. [10] in the analysis of proteins and by Hiraoka and co-workers [11] in the analysis of amino acids. The term WWR ESI was later coined by Mansoori and co-workers [12] to describe this phenomenon. Since then, the WWR ionization phenomenon has been evaluated in number of studies, showing improved detection sensitivities in most cases when used for quantification of small molecules. Tso and Aga [13] reported improved signal to noise ratios for [M + H]^+^ ions in a matrix when using basic mobile phases modified with NH_4_OH for the detection of tetracyclines and sulfonamides. Similarly, improved sensitivity by a reduction in background noise was reported in screening for doping substances in positive ionization mode under basic conditions, also with the use of NH_4_OH as modifier [14].

Sample preparation by solid phase extraction (SPE) is the most commonly used sample pretreatment method for the extraction of ARVDs from wastewater effluent, wastewater influent, surface water, and groundwater [4]. The most frequently used are Oasis® HLB and ISOLUTE® ENV + cartridges, both extracting ARVDs based on polarity [4, 15–18]. In addition, due to the ionizability of the functional groups, i.e., amides, imides, phenols, primary, and tertiary amine groups, ARVDs have also been extracted using mixed mode ion exchange. More specifically, lamivudine (3TC), nevirapine (NVP), ritonavir (RTV), and zidovudine (AZT) have been extracted previously from aqueous solutions using an Oasis® MCX sorbent with recoveries ranging from 73 to 126% [19, 20].

Considering the above, the aim of this study was to reevaluate the optimal ionization mode for selected ARVDs shown in Fig. S1, by evaluating ionization under both acidic and basic chromatographic conditions. Furthermore, to investigate the ionization of these compounds in solution and to determine their extraction recoveries from aqueous samples, a MCX and a MAX sorbents were used.

## Materials and methods

### Chemicals

Efavirenz (EFV), 8-hydroxy efavirenz (8-EFVM), emtricitabine (FTC), nevirapine (NVP), and ritonavir (RTV) were purchased from Cayman Chemicals (Ann Arbor, MI, USA). Lamivudine (3TC), zidovudine (AZT), zidovudine glucuronide (AZTG), and 12-hydroxy nevirapine (NVPM) were obtained from ClearSynth (Mumbai, India). The remaining three analytical standards, 8,14-dihydroxy efavirenz (8,14-EFVM), and desthiazolylmethyloxycarbonyl ritonavir (RTVM) were purchased from Toronto Research Chemicals (Canada). HPLC-grade acetonitrile (ACN) and methanol (MeOH) were purchased from Romil (Waters™ (Microsep), Johannesburg, Gauteng, South Africa). FA and NH_4_OH were obtained from Merck (Darmstadt, Hessen, Germany), and deionized water was obtained using a Milli-Q water purification system (Millipore, Milford, MA, USA).

### Preparation of stock solutions and working solutions

Table S1 provides a summary of the solvents used to prepare each stock solution, and the volumes spiked into a 100 mL working solution of 20% aqueous methanol to improve the poor solubility of hydrophobic ARVDs. The final working concentration of each ARVD ranged from 0.31 to 0.32 μg/mL.

### Direct injections (without column)

A 20 μL aliquot of each ARVD stock solution was diluted 20 times with 50% aqueous acetonitrile containing 0.1% FA (pH 2.5) or 5 mM NH_4_OH (pH 10.7). From each of those dilutions, 10 μL was individually injected using the same dilution solvents as mobile phase at a flow rate of 200 μL/min. An MS scan was performed with a range of 100 to 1000 amu in both the positive and negative ionization mode.

### Sample extraction

Oasis® MCX (6 mL, 150 mg, 30 μm, 1 meq/g) and Oasis® MAX (6 mL, 150 mg, 30 μm, 0.25 meq/g) exchange sorbents from Waters™ via Microsep (Johannesburg, Gauteng, South Africa) were used for sample extraction. Figure S2 illustrates the generic extraction protocol (Oasis®) used to determine the extraction recovery. Briefly, following the equilibration and conditioning steps with MeOH and H_2_O, respectively, the samples were acidified with 2% aqueous FA or basified with 5% aqueous NH_4_OH, as required, before loading onto the respective MCX and MAX sorbents. Following loading, the MCX and MAX sorbents were first washed with 2% aqueous FA or 5% aqueous NH_4_OH, respectively, followed by a MeOH wash for both sorbents. The compounds were subsequently eluted form the MCX and MAX with 5% methanolic NH_4_OH or 2% methanolic FA, respectively. Samples were then evaporated and reconstituted in 20% MeOH for LC–MS analysis.

### Liquid chromatography-mass spectrometry analysis

Analyses were performed on an Agilent 1260 chromatography system equipped with an autosampler, solvent manager, and column oven. Separations were performed using a Kinetex® EVO C_18_ (1.7 μm, 50 × 2.1 mm) column kept at 30 °C. The acidic mobile phase A and B comprised 0.1% FA acid in both H_2_O and ACN, whereas the basic mobile phase A and B consisted of 5 mM NH_4_OH in both H_2_O and ACN. A single mobile phase gradient was used for both ionization modes starting with 1% B and maintaining it for 1 min. B was then increased from 1 to 90% in 16 min, followed by re-equilibration for 5 min at 99% A for a total analysis time of 22 min. The flow rate was 0.4 mL/min, and the injection volume was 2 μL.

The LC system was connected to an Agilent MSD/XT single quadrupole mass spectrometer with an atmospheric jet stream ESI (AJS-ES) source. The capillary and nozzle voltages were 1.5 kV and 1 kV, respectively, for both positive and negative ionization modes. The sheath gas temperature, sheath gas flow, drying gas temperature, drying gas flow, and nebulizer pressure were 300 °C, 12 L/min, 320 °C, 5 L/min, and 34 PSIG. The in-source fragmentation voltage was optimized for each ARVD.

## Results and discussion

**Fig. 1:**
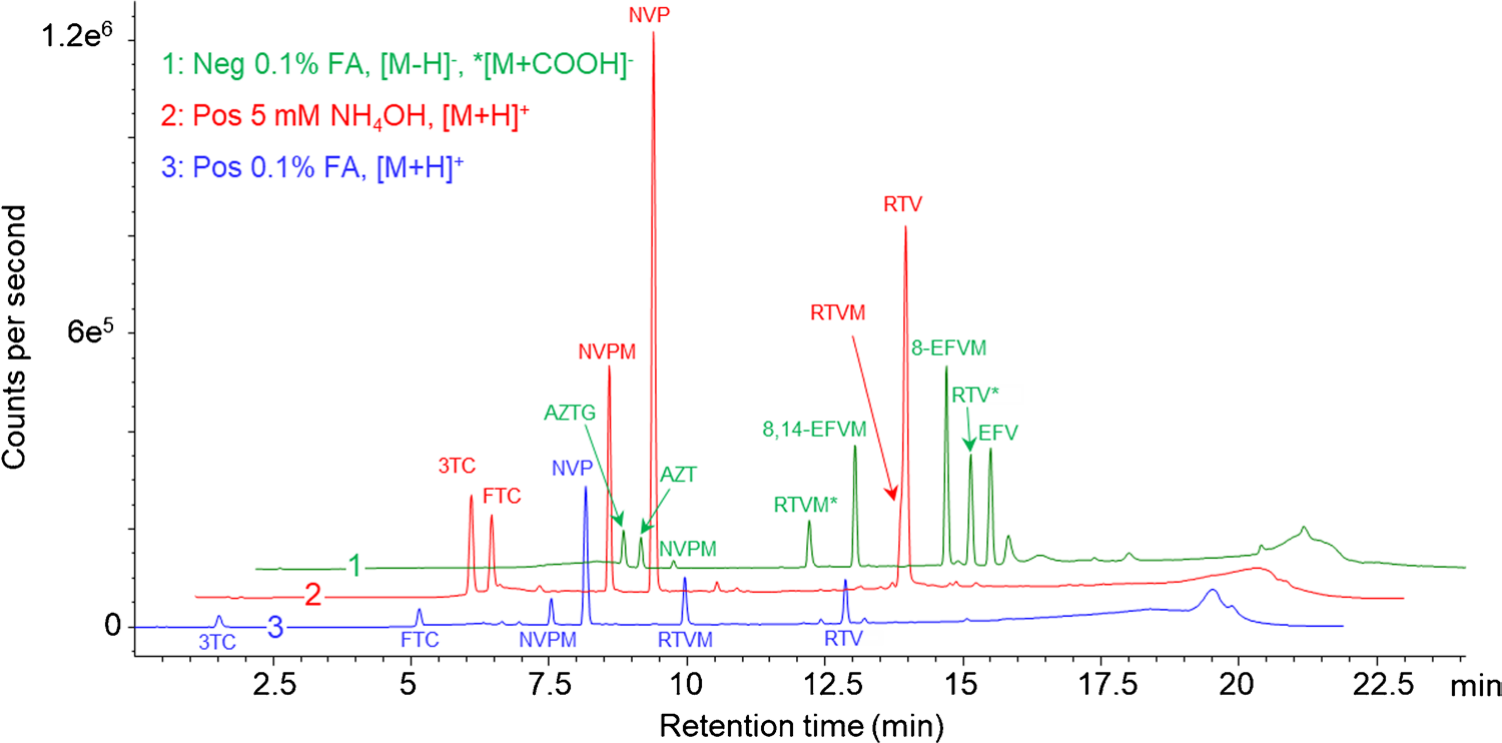
An overlay of the resulting chromatograms generated in the positive and negative ionization mode under acidic (0.1% FA) and basic (5 mM NH_4_OH) LC conditions

Initially, each ARVD was injected (without a column) in a mobile phase containing either 0.1% FA or 5 mM NH_4_OH in aqueous ACN (1:1, v/v) to determine their respective positive and negative ions. The results are summarized in Table S2, which shows the [M + H]^+^ and [M-H]^−^ ions in the positive and negative mode, respectively, for most of the ARVDs. However, RTV and its metabolite, RTVM, produced a predominate formate adduct [M + COOH]^−^ in the negative ion mode using 0.1% FA as modifier. Furthermore, 8,14-EFVM produced no [M + H]^+^ peak, but instead produced low-abundance sodium [M + Na]^+^ and ammonium [M + NH_4_]^+^ adducts under acidic and basic conditions, respectively. Following this, a reversed phase LC–MS method was developed for both polarities under acidic and basic conditions using a Kinetex® EVO C_18_ column, due to its high pH stability. The *m/z* ratios used in the positive and negative ion mode are shown in Table S3. An overlay of the resulting chromatograms is presented in Fig. 1. The chromatogram produced in the negative ion mode using 5 mM NH_4_OH as modifier was omitted due to a high background noise (data not shown).

Comparing the resulting retention times revealed an overall increase in retention of the ARVDs under basic conditions. This was especially true for the early eluting 3TC that showed a threefold increase in the retention factor. However, under basic chromatographic conditions, co-elution of RTV and RTVM was observed, in contrast to what was seen under acidic conditions when these compounds were resolved.

**Fig. 2:**
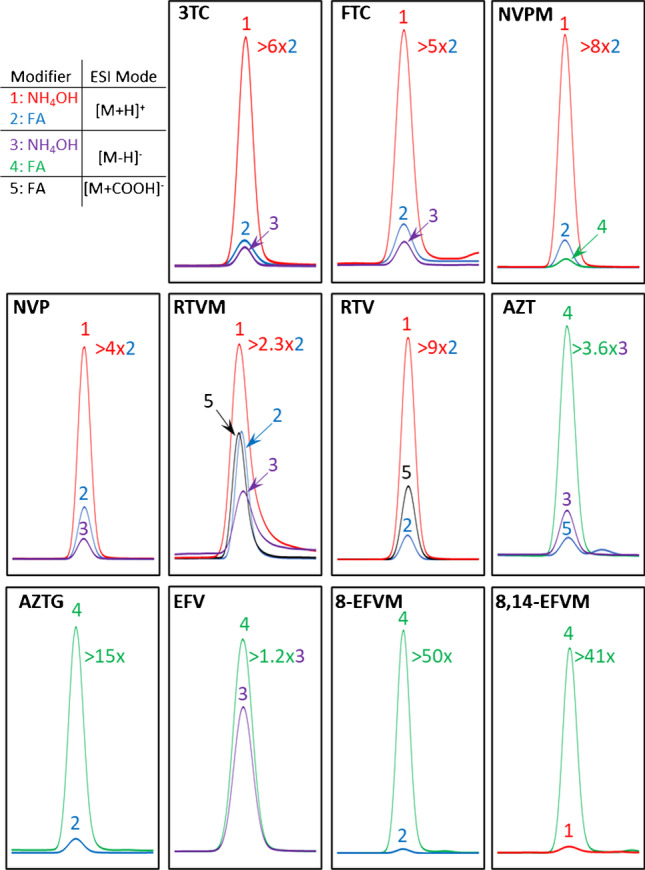
An overlay of the extracted ion chromatograms for each ARVD obtained in the positive and negative ionization mode using 5 mM NH_4_OH and 0.1% FA as mobile phase modifiers

To visually inspect the differences in ionization intensity (peak area) between the four different chromatographic runs, extracted ion chromatographic data for each ARVD were exported to Excel for retention time alignment. The results are illustrated in Fig. 2, which shows an overlay of the extracted ion chromatograms obtained under acidic and basic conditions in the positive and negative ionization mode. The top left-hand corner of Fig. 2 shows the peak numbers (also shown in color) for both ionization modes under acidic and basic mobile phase conditions. Furthermore, note that the fold increase (> #x) shown in the top right-hand corner of each tile is only shown for the WWR ESI, i.e., using the same ionization mode under different LC conditions. For example, 3TC has a sixfold increase in peak area using NH_4_OH compared to FA in the positive ionization mode. It is evident from these results that under basic conditions, 3TC, FTC, NVP, NVPM, RTV, and RTVM yielded a 2.3- to ninefold increase in peak area in the positive ionization [M + H]^+^ mode using 5 mM NH_4_OH as modifier when compared with positive ion [M + H]^+^ detection under acidic conditions. Similarly, the most intense signal for AZT, AZTG, EFV, 8-EFVM, and 8, 14-EFVM was obtained under acidic conditions operating in the negative ionization mode. In particular, AZT and EFV revealed a 1.2- and 3.6-fold increase, respectively, in the signal intensity, whereas no [M-H]^−^ ions were detected under basic conditions for AZTG, 8-EFVM, and 8,14-EFVM. As already mentioned previously, this phenomenon of intense [M + H]^+^ ion formation under basic LC conditions and intense [M-H]^−^ ion formation under acidic LC conditions is known as the WWR ESI, since it does not follow the conventional ionization of compounds in solution [12]. Several theories have been proposed to explain the protonation of compounds under basic conditions during the ESI process. The most likely theory is that gas phase NH_4_^+^ ions are produced during the electrospray process, which serve as a proton donor when NH_4_OH is used as modifier [13]. Using sulfonamides as a model compound, this theory was deduced from the observation that basic modifiers other than NH_4_OH, i.e., sodium hydroxide and tetramethylammonium hydroxide produced no [M + H]^+^ peak, proposing that a basic modifier with an ionizable gas phase proton is necessary [13]. On the other hand, the production of [M-H]^−^ ions under acidic conditions could be explained by the gas-phase proton affinity of the formic acid anions [21].

In comparison, previous studies predominately used formic acid and less frequently used ammonium formate or acetate as modifier in the positive ionization mode for the quantification of 3TC [22, 23], FTC [23, 24], RTVM [23], RTV [16, 20, 23, 25, 26], NVP [22, 23], NVPM [23], AZT [15, 17, 22, 23, 25–27], and EFV [16, 23, 25, 26] in aqueous samples. On the other hand, one study employed negative ionization using acetic acid as modifier for the quantification of AZT and EFV [18]. Based on our results, using NH_4_OH as modifier in the positive ionization mode outperforms FA in both ionization modes by several factors in the case of 3TC, FTC, NVP, NVPM, RTV, and RTVM. In addition, using FA in the negative ionization mode vastly outperformed FA or NH_4_OH in the positive ionization mode and NH_4_OH in the negative ion mode for AZT, AZTG, EFV, 8-EFVM, and 8,14-EFVM. No [M + H]^+^ peak was detected for EFV or 8,14-EFVM in this study, irrespective of the mobile phase pH.

The frequent use of FA for the detection of ARVDs in the positive ionization mode can be attributed to various factors. Firstly, as already mentioned, ARVDs containing amides, primary and tertiary amines are considered weakly basic compounds, and therefore, it is assumed that the addition of an acid in solution will enhance the subsequent ESI process [5, 8, 28]. Secondly, concern over the stability of earlier silica-based LC columns that can undergo hydrolysis at high mobile phase pH values made the use of acidified mobile phases commonplace. However, more recently, many manufactures produce columns that are stable at high pH values.

**Fig. 3:**
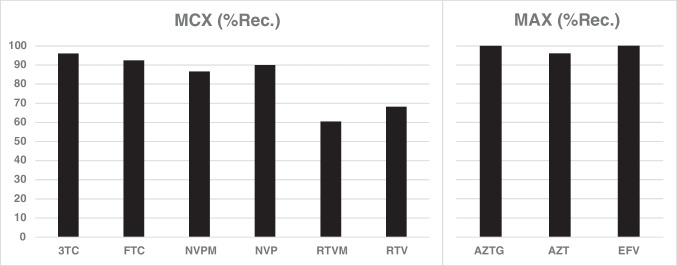
Extraction recoveries of ARVDs from 20% aqueous methanol using MCX and MAX sorbents

To demonstrate that the ions produced in solution do not necessarily reflect the nature of the ions produced during the ESI process, MCX and MAX extraction sorbents were used. For experimental details refer to the “Sample extraction” section and Fig. S2. For maximum retention of ARVDs on a MCX or MAX sorbent, the working solution was either acidified with 2% FA or basified with 5% NH_4_OH, respectively. Following the respective washing steps, the ARVDs were desorbed from the MCX and MAX sorbent using 5% NH_4_OH and 2% FA, respectively. The results are illustrated in Fig. 3. This shows that 3TC, FTC, NVPM, NVP, RTVM, and RTV are highly protonated in acidic (2% FA) solution, yielding recoveries ranging from 60 to 95% when using MCX sorbents. On the other hand, AZT, AZTG, and EFV yielded recoveries > 96% using a MAX sorbent under basic conditions, pointing to the formation of deprotonated ARVDs under basic conditions.

The extraction of 8-EFVM and 8,14-EFVM using a MAX sorbent revealed the production of a second LC peak for both at an increased retention time, illustrated in Fig. S3a (asterisk). Consequently, determining the extraction recoveries for these analytes were not possible. To determine the cause of this anomaly, the 2 mL working solution was mixed with either 4 mL 5% aqueous NH_4_OH to mimic the loading and washing steps or 4 mL 2% methanolic FA used for the final elution step. The samples were then left standing for approximately 10 min (duration of a MAX extraction) at room temperature (23 °C) before injection. The 5% aqueous NH_4_OH dilution were injected directly, whereas the 2% methanolic FA eluate was diluted with water (1:4, v/v) to make it amenable for reversed phase LC. Furthermore, to determine if heating during the final evaporation step at 50 °C caused the formation of double peaks, the final eluate, 2% methanolic FA, was also diluted (1:4, v/v) with water and injected without the evaporation step. Figure S3b represents the chromatogram of the working solution prior to extraction. Temperature was not the cause since the same LC results were obtained before and after evaporation illustrated in Fig. S3a. Furthermore, the 2% methanolic FA dilution produced the same results as the working solution shown in Fig. S3b. However, the 5% aqueous NH_4_OH dilution produced the same additional peaks compared to the MAX extraction for both 8,14-EFVM and 8-EFVM at an increased retention time shown in Fig. S3c. This anomaly was not observed for EFV. Despite this, it is clear from Fig. S3a that both 8-EFVM and 8,14-EFVM has a strong affinity for MAX sorbents under basic conditions.

Table 1 summarizes the ARVD ions that are formed in solution in the presence of an acid or base and the preferred ion exchange sorbent yielding the highest recoveries. In addition, the ions formed in highest abundance for each analyte during the ESI process are also shown. It is clear from these results that the equilibrium concentrations of ions in solution do not necessarily reflect the ions produced by electrospray ion droplets, thereby supporting the WWR ESI phenomenon.

**Table 1:**
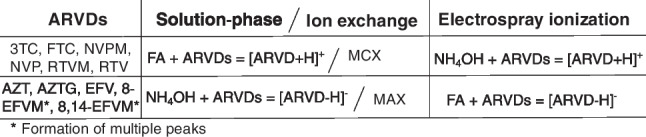
A summary of the ion exchange sorbent used for each ARVD and the preferred modifier for optimal ionization during the ESI process

## Summary

In this study, we have shown improved detection sensitivities by using the WWR ESI for the detection of ARVDs. Using a basic NH_4_OH-modified mobile phase for detection of [M + H]^+^ ions and using an acidic FA-modified mobile phase for detection of [M-H]^−^ ions increased the detection sensitivity multiple fold when compared to the conventional use of acidified mobile phases for the detection of [M + H]^+^ ions. The significant reduction in *m/z* signal intensity observed when FA was used for detection of ARVDs in positive mode, which has been used in most of the previous studies, implies that ARVDs with low abundances can go undetected when analyzing surface waters and wastewater effluent. In addition, by having improved sensitivities, smaller sample volumes can be used for extractions, thereby reducing the amount of consumables used. Using the WWR ionization technique can also reduce analysis time, and the use of solvents in that detection of positively and negatively charged ions can be done in a single analysis method using NH_4_OH as a modifier as was seen for the simultaneous detection of antibiotics and estrogens in a single analysis method [13].

We also developed a selective extraction procedure using MCX and MAX sorbents, which yielded extraction recoveries exceeding 60%. Although these extraction recoveries were only determined in MilliQ water, it serves as a good starting point for the extraction of ARVDs from complex samples such as wastewater effluent and aqueous environmental samples. Furthermore, the WWR ionization is further supported when comparing the specific mixed-mode ion exchange sorbent recoveries with the ions produced during the ESI process, proving that pre-ionization of compounds in solution does not necessarily enhance the signal intensity achieved during the ESI process.

## Supplementary Information

Below is the link to the electronic supplementary material.

Supplementary file1 (DOCX 110 KB)

## References

[1] Omotola EO, Genthe B, Ndlela L, Olatunji OS (2021). Environmental risk characterization of an antiretroviral (ARV) lamivudine in ecosystems. Int J Environ Res Public Heal.

[2] Fernández LP, Brasca R, Repetti MR, Attademo AM, Peltzer PM, Lajmanovich RC, et al. Bioaccumulation of abacavir and efavirenz in Rhinella arenarum tadpoles after exposure to environmentally relevant concentrations. Chemosphere. 2022;301. 10.1016/j.chemosphere.2022.134631 35443209

[3] Gupta RK, Gregson J, Parkin N, Haile-selassie H, Tanuri A, Forero LA (2020). HIV-1 drug resistance before initiation or re-initiation of first-line antiretroviral therapy in low-income and middle-income countries: a systematic review and meta-regression analysis. Lancet Infect Dis.

[4] Nannou C, Ofrydopoulou A, Evgenidou E, Heath D, Heath E, Lambropoulou D (2019). Analytical strategies for the determination of antiviral drugs in the aquatic environment. Trends Environ Anal Chem.

[5] Morgan J, Greenberg A, Liebman JF (2012). Paradigms and paradoxes: O- and N-protonated amides, stabilization energy, and resonance energy. Struct Chem.

[6] Fraenkel G, Franconi C (1960). Protonation of amides. J Am Chem Soc.

[7] Bagno A, Scorrano G (1996). Site of ionization of polyfunctional bases and acids. 1. Ab Initio Proton Affinities. J Phys Chem.

[8] Raczyńska ED, Gal JF, Maria PC, Zientara K, Szelag M (2007). Application of FT-ICR-MS for the study of proton-transfer reactions involving biomolecules. Anal Bioanal Chem.

[9] Cech NB, Enke CG (2001). Practical implications of some recent studies in ESI fundamentals. Mass Spectrom Rev.

[10] Kelly MA, Vestling MM, Fenselau CC, Smith PB (1992). Electrospray analysis of proteins: a comparison of positive-ion and negative-ion mass spectra at high and low pH. Org Mass Spectrom.

[11] Hiraoka K, Murata K, Kudaka I (1995). Do the electrospray mass spectra reflect the ion concentrations in sample solution?. J Mass Spectrom Soc Jpn.

[12] Mansoori BA, Volmer DA, Boyd RK (1997). “Wrong-way-round” electrospray ionization of amino acids. Rapid Commun Mass Spectrom.

[13] Tso J, Aga DS (2011). Wrong-way-round ionization of sulfonamides and tetracyclines enables simultaneous analysis with free and conjugated estrogens by liquid chromatography tandem mass spectrometry. Anal Chem.

[14] Virus ED, Sobolevsky TG, Rodchenkov GM (2012). “Wrong-way-round ionization” and screening for doping substances in human urine by high-performance liquid chromatography/orbitrap mass spectrometry. J Mass Spectrom.

[15] Ngumba E, Gachanja A, Nyirenda J, Maldonado J, Tuhkanen T (2020). Occurrence of antibiotics and antiretroviral drugs in source-separated urine, groundwater, surface water and wastewater in the peri-urban area of Chunga in Lusaka, Zambia. Water SA.

[16] Abafe OA, Späth J, Fick J, Jansson S, Buckley C, Stark A (2018). LC-MS/MS determination of antiretroviral drugs in influents and effluents from wastewater treatment plants in KwaZulu-Natal, South Africa. Chemosphere.

[17] Peng X, Wang C, Zhang K, Wang Z, Huang Q, Yu Y (2014). Profile and behavior of antiviral drugs in aquatic environments of the Pearl River Delta, China. Sci Total Environ.

[18] Horn S, Vogt T, Gerber E, Vogt B, Bouwman H, Pieters R (2022). HIV-antiretrovirals in river water from Gauteng, South Africa: Mixed messages of wastewater inflows as source. Sci Total Environ.

[19] Aminot Y, Le Menach K, Pardon P, Etcheber H, Budzinski H (2016). Inputs and seasonal removal of pharmaceuticals in the estuarine Garonne River. Mar Chem.

[20] Aminot Y, Litrico X, Chambolle M, Arnaud C, Pardon P, Budzinski H (2015). Erratum to: Development and application of a multi-residue method for the determination of 53 pharmaceuticals in water, sediment, and suspended solids using liquid chromatography-tandem mass spectrometry (Analytical and Bioanalytical Chemistry 10.1007/s00. Anal Bioanal Chem.

[21] Wu Z, Gao W, Phelps MA, Wu D, Miller DD, Dalton JT (2004). Favorable effects of weak acids on negative-ion electrospray ionization mass spectrometry. Anal Chem.

[22] Prasse C, Schlüsener MP, Schulz R, Ternes TA (2010). Antiviral drugs in wastewater and surface waters: a new pharmaceutical class of environmental relevance?. Environ Sci Technol.

[23] Mosekiemang TT, Stander MA, de Villiers A (2019). Simultaneous quantification of commonly prescribed antiretroviral drugs and their selected metabolites in aqueous environmental samples by direct injection and solid phase extraction liquid chromatography - tandem mass spectrometry. Chemosphere.

[24] Mlunguza NY, Ncube S, Mahlambi PN, Chimuka L, Madikizela LM (2020). Determination of selected antiretroviral drugs in wastewater, surface water and aquatic plants using hollow fibre liquid phase microextraction and liquid chromatography - tandem mass spectrometry. J Hazard Mater.

[25] Yao L, Dou WY, Ma YF, Liu YS (2021). Development and validation of sensitive methods for simultaneous determination of 9 antiviral drugs in different various environmental matrices by UPLC-MS/MS. Chemosphere.

[26] Wood TP, Duvenage CSJ, Rohwer E (2015). The occurrence of anti-retroviral compounds used for HIV treatment in South African surface water. Environ Pollut.

[27] Muriuki C, Kairigo P, Home P, Ngumba E, Raude J, Gachanja A (2020). Mass loading, distribution, and removal of antibiotics and antiretroviral drugs in selected wastewater treatment plants in Kenya. Sci Total Environ.

[28] Tan A, Fanaras JC (2019). Use of high-pH (basic/alkaline) mobile phases for LC–MS or LC–MS/MS bioanalysis. Biomed Chromatogr.

