# Supplementary material for: Evaluating the “wrong-way-round” electrospray ionization of antiretroviral drugs for improved detection sensitivity
Source: Anal Bioanal Chem. 2023 Jan 13;415(6):1187–93. doi: 10.1007/s00216-022-04499-1 (PMC9899738; doi:10.1007/s00216-022-04499-1)
Supplement: Supplementary file 1 — Supplementary file1 (DOCX 110 KB) [file 216_2022_4499_MOESM1_ESM.docx]

Supplementary Information

**Evaluating the “wrong-way-round” electrospray ionization of antiretroviral drugs for improved detection sensitivity**

Pieter Venter, Rianita van Onselen

Biomedical Research and Innovation Platform, South African Medical Research Council, P.O. Box 19070, Tygerberg 7505, South Africa

**
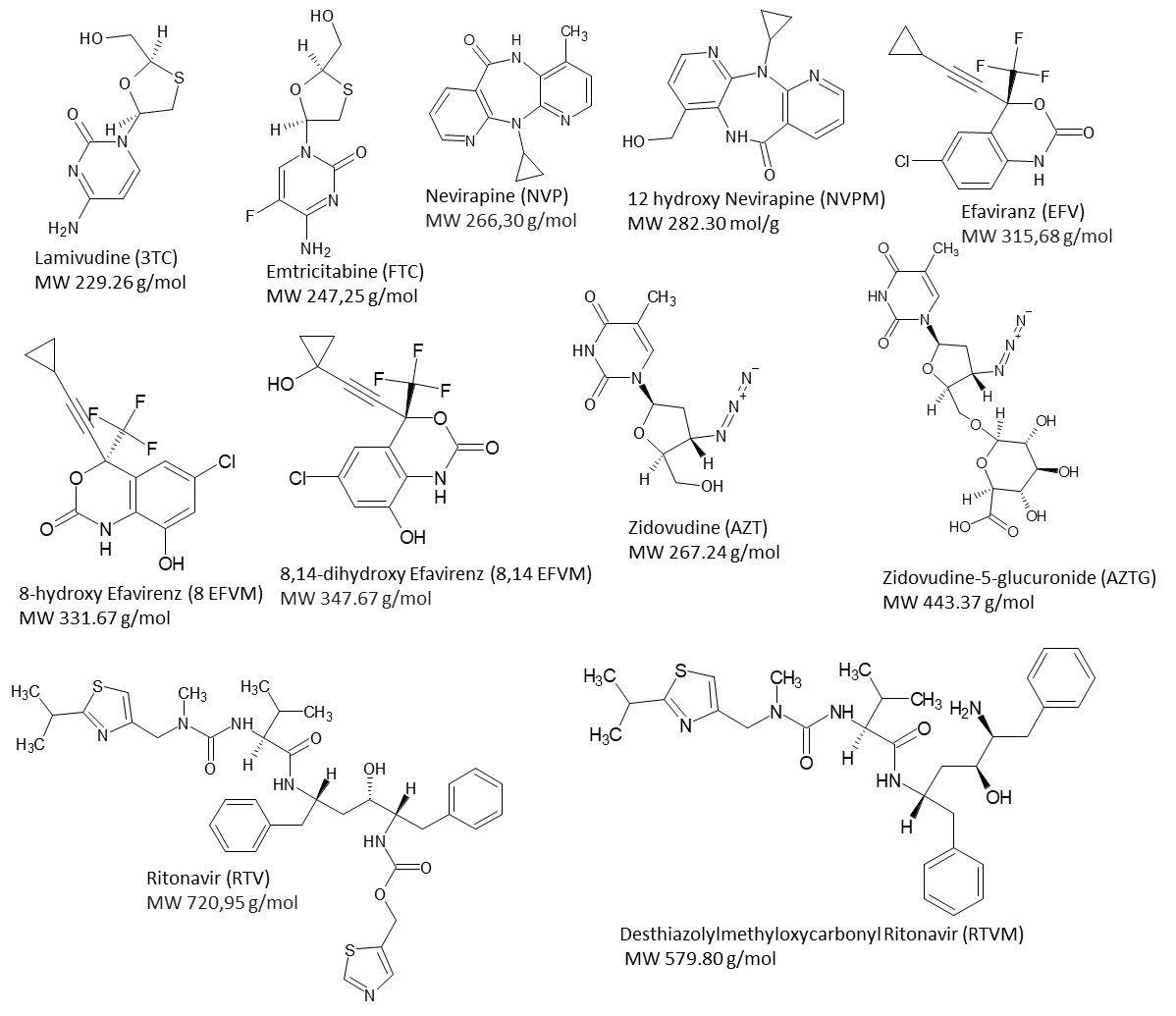
**

**Fig S1:** Structures and molecular weights of the ARVDs under investigation in this study.

**Table S1**: Preparation of stock solutions and the working solution in 20% aqueous methanol

| **ARVDs** | **Solvent used to prepare stock solution** | **Stock solution concentration (mg/mL)** | **Volume spiked (μL) into 100 mL H_2_O/MeOH, 4:1, v/v** | **Working concentration (μg/mL)** |
| --- | --- | --- | --- | --- |
| Ritonavir | MeOH | 0.9 | 36 | 0.32 |
| Nevirapine | MeOH | 2.9 | 11 | 0.32 |
| Efavirenz | MeOH | 1.3 | 25 | 0.32 |
| Lamivudine | H_2_O | 4.6 | 7 | 0.32 |
| Zidovudine | MeOH | 2.5 | 13 | 0.32 |
| Zidovudine glucuronide | (1:1 MeOH/H2O) | 5.0 | 6.5 | 0.32 |
| 12-hydroxy Nevirapine | MeOH | 1.4 | 23 | 0.32 |
| Emtricitabine | H_2_O | 2.3 | 14 | 0.32 |
| 8,14-dihydroxy Efavirenz | MeOH | 1.0 | 32 | 0.32 |
| 8-hydroxy Efavirenz | MeOH | 1.0 | 32 | 0.32 |
| Desthiazolylmethyloxycarbonyl Ritonavir | DMSO | 1.5 | 21 | 0.31 |

**
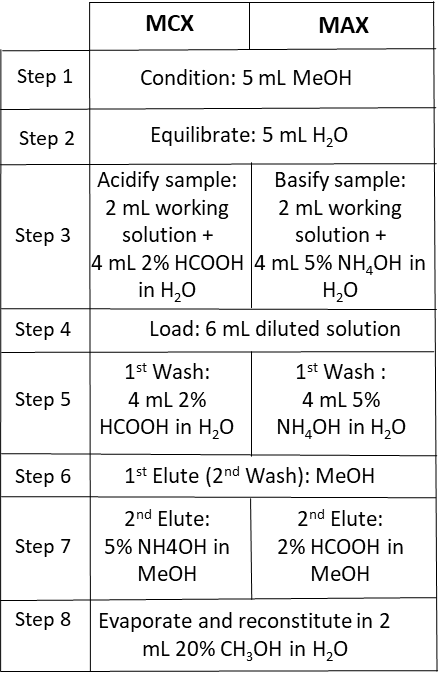
**

**Fig S2:** A flow diagram of the MCX and MAX extraction procedures.

**Table S2:** Ion production in the positive and negative ionization mode under acidic (0.1% FA) and basic (5 mM NH_4_OH) conditions.

| **ARVD** | **MW** | **[M+H]^+^ in 0.1% FA** | **[M-H]^-^ in 0.1% FA** | **[M+H]^+^ in 5mM NH_4_OH** | **[M-H]^-^ in 5mM NH_4_OH** |
| --- | --- | --- | --- | --- | --- |
| 3TC | 229.26 | 230.0 | 228.0 | 230.0 | 228.0 |
| FTC | 247.24 | 248.0 | 246.0 | 248.0 | 246.0 |
| AZTG | 443.37 | 444.1 | 442.1 | 444.1 | 442.1 |
| AZT | 267.24 | 268.1 | 266.1 | 268.1 | 266.1 |
| NVP | 282.3 | 283.1 | 281.1 | 283.1 | 281.1 |
| NVPM | 266.30 | 267.1 | 265.1 | 267.1 | 265.1 |
| RTVM | 579.32 | 580.3 | 624.3 [M+COOH]^-^ | 580.3 | 578.3 |
| 8,14 EFVM | 347.02 | 370.0 [M+Na]^+^ | 346.0 | 365.1 [M+NH_4_]^+^ | 346.0 |
| 8 EFVM | 331.02 | 332.0 | 330.0 | 332.0 | 330.0 |
| RTV | 720.31 | 721.3 | 765.3 [M+COOH]^-^ | 721.3 | 719.3 |
| EFV | 315.03 | 316.0 | 314.0 | 316.0 | 314.0 |

**Table S3**: The positive and negative ionization of ARVDs.

| **ARVDs** | **MW (g/mol)** | **[M+H]^+^** | **[M-H]^-^** |
| --- | --- | --- | --- |
| Lamivudine | 229.26 | 230.0 | 228.0 |
| Emtricitabine | 247.25 | 248.0 | 246.0 |
| Zidovudine-5-glucuronide | 443.37 | 444.0 | 442.0 |
| Zidovudine | 267.24 | 268.0 | 266.0 |
| 12-Hydroxy Nevirapine | 282.30 | 283.0 | 281.0 |
| Nevirapine | 266.30 | 267.0 | 265.0 |
| Desthiazolylmethyloxycarbonyl Ritonavir | 579.80 | 580.1 | 624.0 |
| 8,14-Dihydroxy Efavirenz | 347.67 | 348.0 | 346.0 |
| 8-Hydroxy Efavirenz | 331.67 | 332.0 | 330.0 |
| Ritonavir | 720.90 | 721.3 | 765.0* |
| Efavirenz | 315.67 | 316.0 | 314.0 |

*Formate adduct [M+COOH]^-^


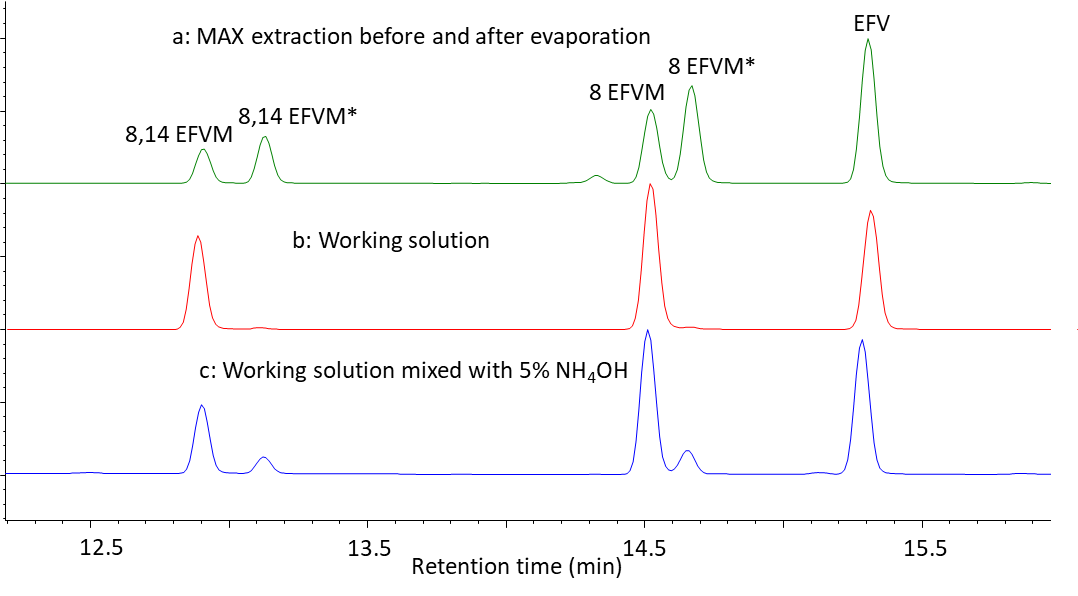


**Fig S3**: The formation of multiple peaks (indicated with an asterisk) following a MAX extraction for 8,14-EFVM and 8-EFVM measured in the negative ionization mode using FA as modifier. LC trace a, b, and c represents the MAX extraction, working solution and working solution mixed with 5% NH_4_OH, respectively.
